# Artificial Intelligence in Chronic Coronary Syndromes: Linking Cellular Pathways to Precision Risk Prediction

**DOI:** 10.3390/cells15212011

**Published:** 2026-11-05

**Authors:** Ibrahim Antoun, Georgia R. Layton, Riyaz Somani, Giovanni Mariscalco, G. André Ng, Mustafa Zakkar

**Affiliations:** 1 Division of Cardiovascular Sciences, University of Leicester, Leicester LE3 9QP, UK; 2 Department of Cardiology, University Hospitals of Leicester NHS Trust, Glenfield Hospital, Leicester LE3 9QP, UK; 3 Department of Cardiac Surgery, University Hospitals of Leicester NHS Trust, Glenfield Hospital, Groby Road, Leicester LE3 9QP, UK; 4 Leicester British Heart Foundation Centre of Research Excellence, Glenfield Hospital, Groby Road, Leicester LE3 9QP, UK; 5 Leicester National Institute for Health and Care Research Biomedical Research Centre, Glenfield Hospital, Groby Road, Leicester LE3 9QP, UK

**Keywords:** chronic coronary syndromes, artificial intelligence, cellular pathways, vascular biology, endothelial injury, microvascular dysfunction, perivascular adipose tissue, inflammation, multi-omics, precision cardiology

## Abstract

Chronic coronary syndromes (CCSs) are no longer defined only by fixed stenosis of the epicardial coronary arteries. CCSs reflect a dynamic biological process. Key contributors include endothelial activation, lipid retention, macrophage foam-cell formation, vascular smooth muscle cell phenotypic switching, and extracellular matrix remodelling. Microvascular rarefaction, perivascular adipose tissue inflammation, cardiometabolic comorbidity, and biological ageing add further complexity. This broader cellular and molecular model provides a rationale for artificial intelligence (AI), because conventional tools often assess anatomy, physiology, inflammation, and systemic biology in isolation. In this narrative review, we examine how AI and machine learning may support a more mechanistic and individualised approach to CCS by linking cellular pathways to measurable clinical signatures. We focus on AI-enabled plaque phenotyping, non-invasive physiology and ischaemia modelling, assessment of coronary microvascular dysfunction, perivascular inflammation mapping, and the integration of multi-omics with cardiometabolic risk profiling. Most of these tools, however, provide imaging-based or data-derived surrogates rather than direct measurements of endothelial activation, inflammation, or calcification biology. Within this limitation, current evidence suggests that AI can quantify subvisual high-risk plaque features, support non-invasive flow assessment, detect imaging patterns associated with microvascular dysfunction and perivascular inflammation, and, in research settings, relate molecular signatures to long-term cardiovascular risk. Clinical adoption remains constrained by retrospective study designs, limited external validation, heterogeneous imaging protocols, gaps in explainability, regulatory uncertainty, and algorithmic bias across sex, ethnicity, age, and resource settings. Future work should prioritise prospective trials, transparent reporting, federated validation, clinically interpretable outputs, and integration with guideline-based decision pathways. The greatest value of AI in CCS is likely to lie in augmenting mechanistic risk stratification and enabling precision prevention for patients whose risk is poorly captured by anatomy alone.

## 1. Introduction

Chronic coronary syndromes (CCSs) describe a broad clinical and biological continuum rather than a single anatomical state. The 2024 European Society of Cardiology guidelines emphasise that patients with suspected or established CCS require evaluation that extends beyond the presence or absence of obstructive epicardial coronary artery disease [1]. Ischaemia and symptoms may arise from plaque burden, endothelial injury, coronary microvascular dysfunction, vasomotor disorders, chronic inflammation, and cardiometabolic disease [2].

This shift matters because traditional risk scores and standard visual interpretation of non-invasive tests often capture only part of the disease process. A patient may have non-obstructive coronary arteries but substantial plaque activity, microvascular dysfunction, or systemic inflammatory risk. Conversely, a visually moderate stenosis may not cause flow limitation. These discordances create diagnostic uncertainty and can lead to both over-investigation and undertreatment.

Artificial intelligence (AI), including machine learning and deep learning, offers a way to combine anatomical, physiological, electrocardiographic (ECG-derived), imaging, and molecular data into clinically useful risk models [3]. It has been applied across many areas of cardiology [4,5,6]. In CCS, relevant AI applications are not limited to detecting stenosis. They include quantitative plaque phenotyping, functional ischaemia modelling, microvascular assessment, perivascular inflammation mapping, biomarker discovery, and personalised prognosis. These applications differ substantially in maturity. AI-assisted CCTA plaque and stenosis quantification and CT-derived physiological modelling have external validation and, in some cases, guideline or regulatory recognition, whereas microvascular assessment, perivascular inflammation mapping, and multi-omics integration remain largely exploratory. Throughout this review, we separate three levels of maturity: clinically usable tools with regulatory clearance or guideline support; externally validated research models that are not yet in routine use; and early, hypothesis-generating work. We indicate which level applies to each application and avoid implying that these are similarly mature. In doing so, we separate three distinct questions that are often conflated: whether a tool has been externally validated, whether it has regulatory authorisation, and whether it has shown benefit on clinical outcomes. This review synthesises these domains and highlights how AI may help translate cellular pathways, molecular signalling, cardiometabolic comorbidity biology, and vascular inflammation into precision cardiology.

We recognise that the conceptual shift in CCS from the vulnerability of an individual lesion to the vulnerability of the whole patient predates AI and that many high-risk patients (for example, those with established cardiometabolic disease) can be identified by clinical assessment alone and treated with intensive lifestyle and pharmacological prevention. The argument of this review is therefore deliberately narrow. AI is not proposed as a means of recognising patients who are already obviously high risk, nor as a replacement for guideline-based prevention. Its potential incremental value lies in three areas that are difficult to address with clinical judgement alone: quantifying subclinical and subvisual disease burden reproducibly and at scale; detecting residual biological risk in patients who are not flagged by conventional risk factors or who have non-obstructive disease; and integrating heterogeneous imaging, physiological, and molecular signals into a single, auditable estimate of risk. We also acknowledge that several of the modalities discussed are resource-intensive and unequally available, so that their near-term benefit will be uneven; we return to access and equity as an explicit implementation priority (Section 4).

## 2. Review Approach and Scope

This article is a narrative, non-systematic review. It aims to synthesise mechanistic and clinical evidence on AI in CCS rather than provide a quantitative pooled analysis. We searched PubMed/MEDLINE, Embase, Scopus, and Web of Science for English-language articles published up to October 2026, using combinations of the terms “artificial intelligence”, “machine learning”, “deep learning”, “radiomics”, “chronic coronary syndrome”, “coronary CT angiography”, “fractional flow reserve”, “perivascular adipose tissue”, “coronary microvascular dysfunction”, and “multi-omics”. Priority was given to prospective studies, externally validated models, multicentre cohorts, guideline documents, and consensus statements; smaller single-centre and proof-of-concept studies were included when they illustrated an emerging mechanism or application and are identified as such in the text. Because the review discusses the performance, validation, and implementation of clinical AI, we used contemporary AI reporting and evaluation frameworks to inform a qualitative assessment, namely TRIPOD+AI, which is a reporting guideline for prediction models, and DECIDE-AI, which addresses early-stage clinical evaluation [7,8]. These frameworks are not quality-appraisal instruments; we therefore used them only as a reference point and note, where relevant, that many studies report incompletely or lack external validation and prospective evaluation. The review was not registered and did not apply a formal risk-of-bias scoring system, consistent with its narrative scope.

## 3. Applications of AI in Chronic Coronary Syndromes

### 3.1. Pathophysiological Framework for AI in CCS

A useful AI framework for CCS should start from disease biology. Atherosclerosis develops through endothelial activation, lipid retention, immune cell recruitment, macrophage foam-cell formation, smooth muscle cell phenotypic switching, extracellular matrix remodelling, calcification, and fibrous cap disruption. These processes evolve under the influence of diabetes, obesity, chronic kidney disease, hypertension, smoking, ageing, and systemic inflammation [9].

Microvascular dysfunction adds another layer of complexity. Coronary arterioles and capillaries regulate most coronary resistance, yet they remain invisible to routine angiography. Structural remodelling, capillary rarefaction, endothelial dysfunction, impaired nitric oxide bioavailability, and abnormal vasoconstrictor responses can produce ischaemia even in the absence of epicardial stenosis [10]. This mechanism is particularly relevant in ischaemia with non-obstructive coronary arteries (INOCA) and in women, patients with diabetes, and older adults.

AI can help because these mechanisms leave measurable signatures across multiple modalities. Plaque composition appears on coronary computed tomography angiography (CCTA). Vascular inflammation changes pericoronary adipose tissue (PCAT) attenuation. Microvascular impairment alters myocardial perfusion and may influence electrocardiographic patterns [11,12]. Proteomic and metabolomic profiles capture systemic inflammatory and metabolic pathways. The central challenge is to integrate these signals without losing clinical interpretability.

### 3.2. Cellular and Molecular Pathways as the Substrate for AI in CCS

CCTA has become a central test for suspected CCS because it detects coronary atherosclerosis, excludes obstructive disease with high negative predictive value, and provides information on plaque distribution [13,14]. From a cellular perspective, CCTA-derived plaque features are often interpreted as macroscopic correlates of endothelial activation, lipid retention, macrophage accumulation, smooth muscle cell remodelling, extracellular matrix turnover, and calcification. Manual or semi-automated plaque analysis is time-consuming and operator-dependent. Deep learning models can automate coronary centreline extraction, lumen segmentation, vessel wall segmentation, stenosis grading, and plaque composition analysis [15,16].

Endothelial injury initiates and maintains much of the CCS continuum. Reduced nitric oxide bioavailability, oxidative stress, upregulation of adhesion molecules, glycocalyx disruption, and abnormal vasomotor signalling promote leukocyte recruitment and impair coronary flow regulation [17]. These processes are not measured directly in routine imaging. At most, AI can detect imaging or biomarker patterns that are statistically associated with endothelial injury, such as plaque burden, perfusion heterogeneity, and perivascular inflammatory signatures; whether such patterns reflect endothelial biology in an individual patient has not been validated and should be treated as a plausible association rather than a demonstrated inference.

Lipid retention and immune activation drive plaque growth and vulnerability. Oxidised low-density lipoprotein (LDL) uptake promotes macrophage foam-cell formation, inflammasome activation, cytokine release, necrotic core expansion, and fibrous cap weakening [18,19]. Quantitative CCTA and radiomics do not visualise macrophages directly in routine care, but they can capture downstream structural correlates, including non-calcified plaque, low-attenuation plaque, positive remodelling, spotty calcification, and pericoronary inflammatory change [20].

Vascular smooth muscle cell phenotypic switching is another central mechanism. Smooth muscle cells can migrate, proliferate, produce extracellular matrix, contribute to fibrous cap formation, or adopt inflammatory and osteochondrogenic phenotypes that promote plaque instability and calcification [21,22]. AI-derived plaque composition and longitudinal plaque progression metrics may therefore be imaging-based surrogates that correlate with extracellular matrix remodelling and calcification. Any link to processes such as plaque healing or destabilisation remains indirect and hypothesis-generating. Confirming that these metrics track the underlying cellular biology will require longitudinal imaging anchored to histological or molecular reference standards.

Microvascular dysfunction links cellular injury to symptoms and prognosis. Endothelial dysfunction, pericyte dysregulation, arteriolar remodelling, capillary rarefaction, mitochondrial dysfunction, and impaired vasodilator reserve can produce ischaemia without obstructive epicardial disease [23,24]. AI-enabled ECG, cardiovascular magnetic resonance (CMR), positron emission tomography (PET), echocardiographic, and perfusion models are best interpreted as detecting functional or imaging correlates of microvascular dysfunction; they do not measure the underlying cellular phenotype, and the link between model output and cellular biology remains an association rather than a validated patient-level inference.

Perivascular adipose tissue provides one of the more direct, though still indirect, links between cellular signalling and non-invasive imaging. Cytokines released from an inflamed coronary wall alter adipocyte differentiation, lipid storage, and tissue water content, producing measurable changes in pericoronary fat attenuation [11,12]. AI and radiomics can extend this concept beyond a single attenuation threshold by extracting spatial, textural, and vessel-specific imaging features that have been associated with inflammatory remodelling [20,25]. Separately, imaging studies have linked pericoronary fat attenuation to markers of disease activity such as coronary 18F-sodium fluoride uptake [26].

This cellular framing also clarifies the role of cardiometabolic comorbidities. Diabetes, obesity, chronic kidney disease, hypertension, and ageing amplify endothelial dysfunction, oxidative stress, immune activation, impaired autophagy, extracellular matrix turnover, and microvascular rarefaction [27]. Whether multi-omics AI can identify which biological axis dominates in an individual CCS patient and thereby guide mechanism-based prevention remains unproven. This expectation is largely extrapolated from other cardiovascular domains, such as the multi-omics dissection of cardiac fibrosis in heart failure [28], rather than demonstrated in CCS.

### 3.3. AI-Guided CCTA Plaque Phenotyping

AI-guided CCTA plaque phenotyping is most informative when imaging features are interpreted as surrogates of plaque biology. Low-attenuation plaque, positive remodelling, non-calcified plaque volume, spotty calcification, and total plaque burden are thought to correspond to processes such as lipid-rich necrotic core formation, macrophage-driven inflammation, extracellular matrix remodelling, vascular smooth muscle cell phenotypic change, and calcification biology [22,25]. Automated extraction of these features can reduce observer dependence and support a more quantitative assessment of high-risk coronary atherosclerosis.

External validation has shown that deep learning-enabled CCTA can quantify plaque volume and stenosis severity rapidly and with close agreement to expert readers and intravascular ultrasound [15]. This is clinically important because total plaque burden, non-calcified plaque, low-attenuation plaque, and adverse plaque features often predict events beyond stenosis severity alone [15]. Automated analysis can therefore shift CCTA from a binary test for obstruction toward a quantitative tool for risk staging and preventive treatment intensification.

Several principles should guide interpretation. First, AI-derived plaque metrics must be linked to clinical decisions, such as lipid-lowering intensification, anti-inflammatory strategies, or follow-up imaging, rather than being reported as isolated technical outputs. Second, threshold-based reporting should avoid false precision because scanner type, reconstruction parameters, contrast timing, heart rate, and segmentation quality can affect plaque quantification. Third, sex-specific and ethnicity-specific calibration matters because plaque burden and event risk are not distributed equally across populations.

### 3.4. AI and Physiology-Based Ischaemia Modelling

Physiology-based ischaemia modelling addresses a complementary pathway: epicardial stenosis interacts with diffuse atherosclerosis, endothelial-dependent vasodilation, arteriolar tone, and microvascular resistance to determine whether a lesion produces ischaemia [29]. The methods used to estimate CT-derived fractional flow reserve (FFRCT) should be distinguished because they are not interchangeable, and not all of them are AI. Conventional FFRCT applies computational fluid dynamics to CCTA anatomy and is the most extensively validated and guideline-referenced form; machine learning CT-FFR instead trains models to approximate the computational fluid dynamics or invasive result, trading mechanistic transparency for speed; and AI is also used upstream for coronary segmentation and workflow automation rather than for the physiological computation itself [30]. Describing all of these as a single AI tool overstates the specific contribution of AI, and the evidence base differs across them.

AI can accelerate and simplify physiological modelling by learning relationships between anatomy, boundary conditions, flow, and pressure gradients. However, clinicians should avoid treating any single computed value as absolute truth. FFRCT depends on assumptions about hyperaemic microvascular resistance. These assumptions may be less reliable in patients with coronary microvascular dysfunction, left ventricular hypertrophy, diffuse disease, prior infarction, or abnormal vasomotor tone [31]. Intermediate values near the clinical decision threshold require integration with symptoms, plaque burden, perfusion data, and microvascular assessment.

The future direction is not a competition between anatomy and physiology. A plausible direction is to combine plaque phenotype, stenosis severity, haemodynamic significance, local inflammation, and patient-level cardiometabolic risk within an integrated decision-support model. Such a model has not yet been built or prospectively validated and remains a research goal rather than a current capability.

### 3.5. AI Assessment of Coronary Microvascular Dysfunction

Coronary microvascular dysfunction is a key mechanism of CCS, especially in patients with persistent angina and no obstructive epicardial disease. At the cellular level, it reflects endothelial dysfunction, reduced nitric oxide signalling, pericyte and smooth muscle dysregulation, capillary rarefaction, oxidative stress, mitochondrial dysfunction, inflammation, and impaired hyperaemic reserve [24]. Current diagnostic standards include invasive coronary function testing and quantitative assessment of myocardial blood flow using PET or CMR. These tests provide mechanistic clarity but are not available in many centres.

The focus here is on the AI models rather than on the established reference tests against which they are compared, and we highlight representative primary studies with their inputs, prediction targets, reference standards, and validation status. For ECG-based assessment, a self-supervised foundation transformer trained on large volumes of unlabelled 12-lead ECGs and fine-tuned against PET, SPECT, and CMR data has been reported to estimate myocardial flow reserve and flag coronary microvascular dysfunction, improving on earlier task-specific models [32]; here, the input is the raw ECG, the prediction target is an imaging-defined flow-reserve phenotype, the reference standard is quantitative perfusion imaging, and the main limitation is the absence of prospective, externally validated outcome data. For multimodal imaging, an explainability-enhanced model (CMVD_MDAS) integrating automated segmentation and feature extraction from echocardiography, CMR, and PET/CT reported high internal discrimination but a markedly lower external area under the curve (approximately 0.79); this drop reflects the external-validation gap typical of single-programme datasets [33]. Deep learning models applied to stress-perfusion CMR or PET take the perfusion image series as input and output quantitative myocardial blood flow or perfusion reserve, with invasive coronary function testing (coronary flow reserve and the index of microcirculatory resistance) as the reference standard; these are more mature for automating quantification but remain largely validated at expert centres, and echocardiographic models estimate coronary flow velocity reserve, usually in the left anterior descending territory [16,23,24]. Across these applications, the recurring limitations are small or single-programme datasets, inconsistent reference standards, limited external validation, and little evidence that acting on the output improves outcomes; we therefore treat AI microvascular assessment as an early research application in which externally validated primary studies remain few.

The clinical promise is high, but the evidence base remains early. Many models require broader validation against invasive physiology or quantitative perfusion reference standards. Studies should report calibration, subgroup performance, clinical utility, and decision-curve analysis. Without this, AI-based microvascular screening may remain technically impressive but clinically uncertain.

### 3.6. Perivascular Adipose Tissue as an Inflammatory Biosensor

Vascular inflammation drives plaque progression and destabilisation. Perivascular adipose tissue, especially pericoronary adipose tissue, acts as a local sensor of vascular inflammation through bidirectional signalling between the vessel wall and surrounding fat. Inflammation inhibits adipogenesis and increases the aqueous content of perivascular fat, which changes attenuation on CCTA.

The perivascular fat attenuation index was developed to quantify these changes and has shown prognostic value for cardiac mortality in the CRISP-CT study [12]. AI and radiomics can extend this concept by automating adipose tissue segmentation and extracting texture features that are invisible to routine visual assessment. These features may identify residual inflammatory risk even when stenosis is mild or conventional risk scores are intermediate.

This domain fits a mechanistic CCS framework particularly well. It connects local cytokine signalling, adipocyte remodelling, vascular inflammation, and clinical prognosis. The main implementation issue is standardisation. Perivascular attenuation is strongly dependent on acquisition parameters (including tube voltage and contrast timing), reconstruction method and kernel, scanner model, vessel selection, segmentation technique, and the specific software implementation; reported thresholds can shift materially when any of these change, which currently limits cross-study comparison and routine deployment. These approaches should also not be treated as interchangeable: the conventional pericoronary fat attenuation index has validated prognostic associations in post hoc analyses of prospective cohorts, whereas standardised attenuation scores and radiomics-based prognostic models are earlier in development, more protocol-dependent, and less externally validated. Before routine deployment, models require reproducible thresholds, external validation, and prospective evidence that reporting inflammatory risk changes treatment and outcomes [11,26].

### 3.7. Multi-Omics, Biomarkers, and Cardiometabolic Comorbidities

CCS risk reflects systemic biology as much as coronary anatomy. Diabetes, obesity, chronic kidney disease, hypertension, and ageing alter lipid metabolism, immune activation, endothelial repair, oxidative stress, and extracellular matrix turnover [34]. Proteomic, metabolomic, transcriptomic, and genomic data can identify disease pathways that are not captured by routine clinical variables.

Machine learning is well suited to multi-omics because it can handle high-dimensional, correlated variables and non-linear interactions. In chronic coronary disease, proteomic studies have identified circulating signatures associated with cardiovascular mortality and recurrent events [35]. Multi-omics models may eventually support earlier risk detection, pathway-specific treatment selection, and drug-target prioritisation.

However, multi-omics AI is not yet ready for routine CCS care. Most models face issues related to batch effects, platform variability, missing data, population selection, interpretability, and cost. A practical near-term approach is to use omics data to refine biological pathways and generate candidate biomarkers, followed by validation using simpler, clinically deployable assays. Figure 1 provides an overview of the proposed AI-enabled mechanistic framework for CCS, linking vascular imaging, perivascular inflammation, multi-omics, cardiometabolic comorbidities, cellular pathways, and clinical outcomes to precision risk prediction.

## 4. Clinical Implementation, Bias, and Explainability

The main barrier to AI in CCS is not model performance in development datasets but safe generalisation. Models trained within a single health system can fail when deployed across different scanners, acquisition protocols, reporting standards, ethnic groups, sex distributions, comorbidity profiles, or disease prevalence [36]. External validation should be mandatory, and prospective impact studies should test whether AI-guided decisions improve patient-centred outcomes [7,8,37]. Where patient data cannot be centralised, federated validation, which evaluates models across institutions without moving the underlying data, provides a privacy-preserving route to multi-site generalisation. Equity of access is also a concern. Several of the modalities discussed (CCTA, PET, CMR, and multi-omics) are unavailable in many settings, and AI tools risk widening rather than narrowing care gaps unless deployment is planned with access in mind. Scalable, low-cost inputs such as the 12-lead ECG are attractive partly because they may help mitigate this disparity.

Implementation should prioritise AI outputs that are linked to a defined biological substrate and a clear clinical action. In CCS, this means connecting plaque phenotype to preventive intensification, physiology models to angiography and revascularisation decisions, microvascular signatures to coronary function testing, perivascular inflammation to residual inflammatory risk, and omics signals to pathway discovery or biomarker validation [38,39].

Explainability also matters. Clinicians do not need every neural network parameter, but they need outputs that map onto clinical reasoning. A useful system should show image quality, segmentation confidence, the anatomical or biological basis for risk classification, uncertainty estimates, and recommended next steps aligned with guidelines [40]. Black-box predictions without auditability are unlikely to gain trust in CCS pathways. Several evidence gaps must be addressed before AI can be integrated safely into CCS pathways. These include limited external validation, retrospective study design, algorithmic bias, weak explainability, data heterogeneity, and poor integration with actionable clinical decisions. Table 1 summarises these barriers and outlines practical solutions, including multicentre validation, prospective impact studies, subgroup performance reporting, uncertainty estimates, standardised data pipelines, and guideline-linked decision points.

Safe deployment depends on more than discrimination. Models should be assessed for calibration, since predicted risks can be systematically too high or too low even when ranking is good; for dataset (distribution) shift and spectrum bias, since performance varies with scanner, protocol, case mix, and disease prevalence; for the handling of missing data and the quality of the reference labels used in training; for subgroup performance across sex, age, ethnicity, and comorbidity; and for model drift after deployment, which requires scheduled monitoring and planned recalibration. These factors determine whether an apparently high-performing model is safe in practice.

## 5. Suggested Clinical Pathway

A practical AI-enabled CCS pathway should begin with guideline-based clinical assessment, symptom phenotype, comorbidity burden, and pre-test likelihood, which determine who should proceed to CCTA. In patients selected for CCTA, AI can quantify stenosis, total plaque burden, plaque composition, adverse plaque features, and perivascular inflammatory signatures. These outputs are indirect, imaging-based correlates of the underlying biology rather than direct measurements of it, and should be reported with image-quality and confidence metrics so that low-quality studies are flagged for expert review rather than acted on automatically.

The outputs should map to explicit actions, integrated with the clinical context rather than applied mechanically. Referral for invasive angiography should not rest on a single number: an abnormal CT-derived fractional flow reserve (using the established ischaemia threshold of FFR or FFRCT at or below 0.80) supports referral, but the decision should also weigh symptom severity and refractoriness, high-risk anatomy (for example, left main or proximal multivessel disease), lesion location, and pre-test likelihood. Borderline values (approximately 0.76 to 0.80), or findings discordant with symptoms or anatomy, warrant additional functional assessment such as stress perfusion imaging or invasive physiology rather than immediate angiography, and intermediate anatomical stenosis (approximately 40 to 70 percent) is a typical trigger for functional evaluation. A high or progressing plaque burden, or a high-risk plaque phenotype, should prompt intensification of preventive therapy in line with guideline risk categories; for example, findings can be summarised within an established framework such as CAD-RADS 2.0 (which pairs stenosis severity with a plaque-burden modifier) or the coronary artery calcium score so that a patient reclassified upward by extensive non-calcified or low-attenuation plaque becomes a candidate for more intensive lipid-lowering and tighter LDL targets [1,38]. We stress that specific quantitative plaque thresholds are not yet standardised, that no AI-specific treatment rule has been established, and that these metrics should support, not replace, guideline-based risk assessment. In patients with persistent symptoms and non-obstructive disease, AI-supported perfusion or ECG tools may raise suspicion of coronary microvascular dysfunction, but a positive screen should be confirmed by quantitative perfusion (PET or CMR) or by invasive coronary function testing before it changes management. Biomarker and multi-omics data remain a research input and should not drive individual treatment decisions at present.

Several safeguards are essential. Poor image quality, discordance between an AI output and the clinical picture, or a biologically implausible result should trigger human review and override rather than automated action, and the degree of model uncertainty should be shown to the clinician. The major AI applications across CCS domains, their biological substrates, potential clinical value, evidence maturity, and limitations are summarised in Table 2. This framework preserves clinical judgement: AI should support decisions on diagnosis, prevention, revascularisation referral, microvascular testing, and follow-up intensity and should not replace patient assessment, shared decision-making, or invasive testing when symptoms and risk remain discordant.

## 6. Conclusions

AI has the potential to reshape CCS care by moving assessment from isolated stenosis grading to integrated biological risk prediction. The strongest evidence currently supports AI-assisted CCTA plaque quantification and, for non-invasive physiology, computational-fluid-dynamics CT-derived fractional flow reserve in particular, rather than all AI-based implementations. Work in microvascular dysfunction, perivascular inflammation, and multi-omics is earlier and largely hypothesis-generating; these approaches may, in time, connect biological mechanisms to measurable clinical risk, but current evidence in CCS does not yet establish that link.

For AI to become clinically useful in CCS, future studies must demonstrate more than technical accuracy. They must show improved calibration across populations, transparent model behaviour, reproducible performance across platforms, and measurable benefit in clinical decision-making. The goal is not automated cardiology. The goal is better phenotyping, earlier prevention, and more precise care for patients whose risk cannot be explained by coronary anatomy alone. AI should be positioned as a complement to established, widely accessible prevention, adding most where conventional clinical assessment leaves risk unexplained. The evidence currently supports AI for CCTA-based plaque and stenosis quantification and for selected CT-derived physiology, whereas its application to microvascular, perivascular, and multi-omics phenotyping remains investigational, a boundary we have kept explicit rather than presenting these approaches as equivalent.

## Figures and Tables

**Figure 1:**
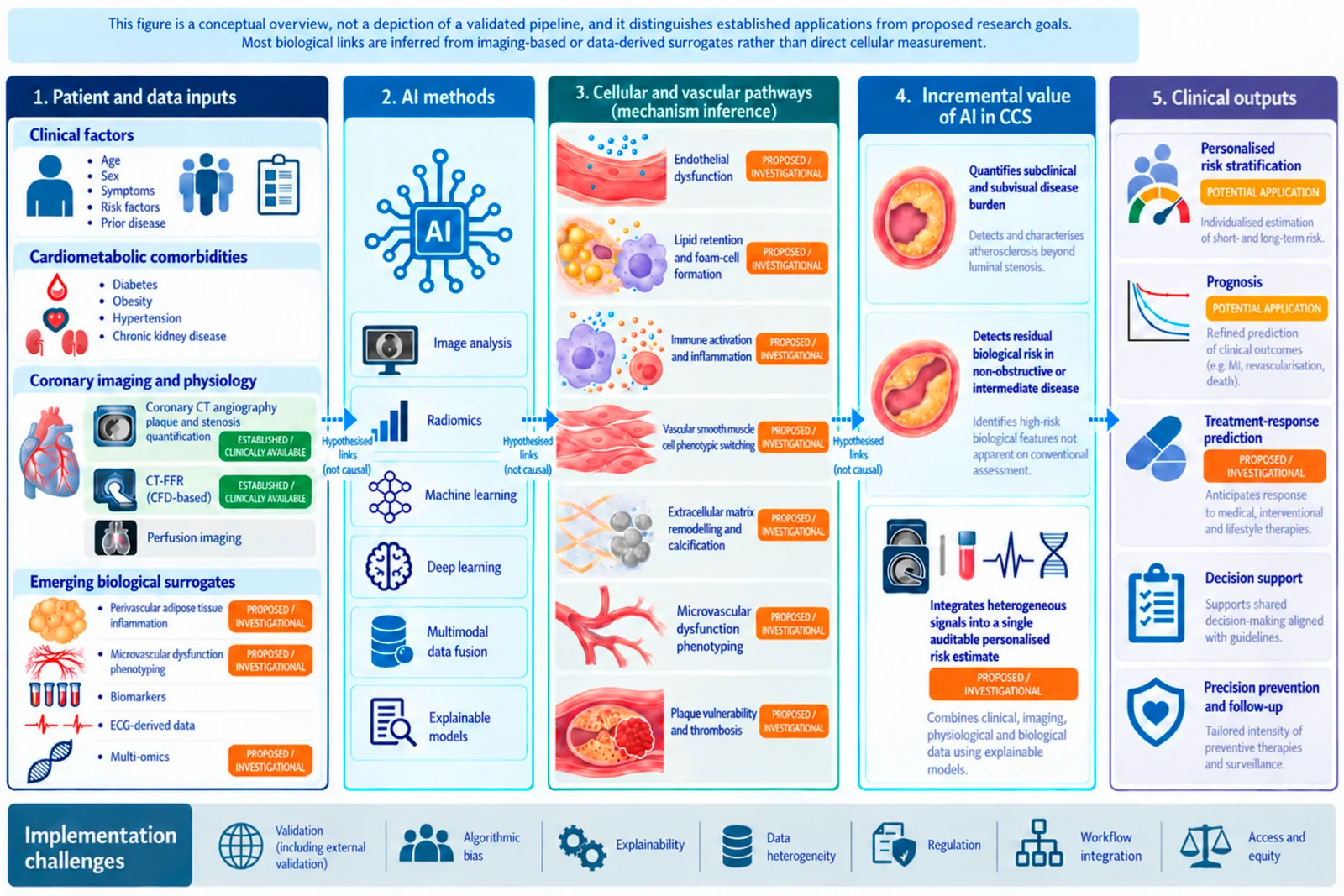
Conceptual framework for artificial intelligence-enabled precision risk prediction in chronic coronary syndromes. This figure is a conceptual overview, not a depiction of a validated pipeline, and it distinguishes established applications from proposed research goals. Established or clinically available elements are AI-assisted CCTA plaque and stenosis quantification and computational-fluid-dynamics CT-derived fractional flow reserve. Proposed or hypothetical elements, shown to indicate direction rather than current capability, include inference of specific cellular pathways, microvascular phenotyping, perivascular and multi-omics integration, and treatment-response prediction. Key challenges include validation, bias, explainability, data heterogeneity, regulation, workflow integration, and equitable access. Green labels denote established or clinically available applications; orange labels denote proposed or investigational applications. The colours indicate evidence maturity, not proof of patient-level cellular inference.Green labels denote established or clinically available applications; orange labels denote proposed or investigational applications. The colours indicate evidence maturity, not proof of patient-level cellular inference.

**Table 1:** Evidence gaps and future directions.

Barrier	Why It Matters in CCS	Recommended Solution
Limited external validation	Models may fail across scanners, sites, ethnic groups, and disease prevalence settings	Use multicentre external validation, transparent reporting, and calibration assessment before deployment
Retrospective study design	High apparent accuracy may not translate into improved clinical outcomes	Conduct pragmatic prospective trials with hard outcomes, workflow endpoints, and cost-effectiveness analysis
Algorithmic bias	Underdiagnosis may occur in women, older adults, minority groups, and low-resource settings	Mandate subgroup reporting and use diverse training and validation cohorts
Weak explainability	Clinicians may not trust predictions that do not map to anatomy or biology	Provide segmentation overlays, uncertainty estimates, feature attribution, and guideline-linked action statements
Data heterogeneity	CCTA, ECG, CMR, PET, and omics pipelines differ across centres	Standardise acquisition, preprocessing, reporting, and quality-control metrics
Clinical integration	AI outputs can add noise if they do not change management	Embed AI in specific decision points, such as preventive intensification, FFRCT referral, or microvascular testing
Poor calibration and dataset shift	Discrimination (for example, AUC) can look high while predicted risks are miscalibrated, and performance degrades under distribution shift across sites, scanners, and populations	Report calibration, not only discrimination; test under external and prospective distribution shift; recalibrate for each deployment setting
Spectrum bias, missing data, label quality, and model drift	Performance varies with case mix and disease prevalence; missing data and noisy reference labels bias training; performance drifts after deployment	Report spectrum and subgroup performance; prespecify missing-data handling; audit label quality; monitor for drift with scheduled re-evaluation

Abbreviations: AI, artificial intelligence; CCSs, chronic coronary syndromes; CCTA, coronary CT angiography; CMR, cardiovascular magnetic resonance; ECG, electrocardiogram; FFRCT, fractional flow reserve derived from CT; PET, positron emission tomography.

**Table 2:** AI applications in chronic coronary syndromes by clinical domain.

Clinical Domain	Cellular or Molecular Pathway	AI Approach	Potential Clinical Value	Key Limitations	Evidence Maturity
CCTA plaque phenotyping	Endothelial activation, LDL retention, macrophage foam-cell formation, VSMC phenotypic switching, extracellular matrix remodelling, calcification, and fibrous cap biology	Deep learning segmentation, quantitative plaque analysis, radiomics	Improves risk stratification beyond stenosis and supports preventive treatment decisions	Scanner variability, segmentation errors, uncertain thresholds for longitudinal change	Externally validated (multicentre); some tools carry regulatory clearance; outcome benefit not yet shown in randomised trials
Physiology-based ischaemia modelling	Epicardial flow limitation interacting with arteriolar tone, endothelial-dependent vasodilation, diffuse disease physiology, and microvascular resistance	Computational modelling, deep learning surrogates, FFRCT workflows	Reduces unnecessary invasive angiography and improves diagnostic yield in selected patients	Assumptions about microvascular resistance, grey-zone values, limited applicability in some complex phenotypes	Computational-fluid-dynamics CT-FFR is externally validated, guideline-referenced, and regulatory-cleared; machine learning CT-FFR and AI segmentation are less validated; outcome-benefit evidence is limited
Microvascular dysfunction	Endothelial dysfunction, reduced nitric oxide bioavailability, capillary rarefaction, pericyte dysfunction, oxidative stress, and impaired hyperaemic reserve	AI-ECG, automated perfusion analysis, multimodal imaging classifiers	May provide scalable screening for INOCA and persistent angina after exclusion of obstructive disease	Requires validation against invasive physiology and quantitative perfusion reference standards	Early research; limited external validation; no regulatory clearance; clinical benefit not demonstrated
Perivascular inflammation	Cytokine signalling between the vessel wall and pericoronary adipocytes, adipocyte dedifferentiation, lipolysis, water-content shift, and inflammatory remodelling	Automated PCAT segmentation, fat attenuation index, CT radiomics	Identifies residual inflammatory risk; any role in tracking response to anti-inflammatory or lipid-lowering therapy is unproven and awaits prospective intervention data	Protocol dependence, need for standardised definitions, limited prospective treatment data	The fat attenuation index has validated prognostic associations (post-hoc prospective data); standardised scores and radiomics models are earlier and protocol-dependent; no regulatory clearance; benefit of acting on it not shown
Multi-omics and biomarkers	Proteomic, metabolomic, transcriptomic, and genetic pathways related to inflammation, lipid metabolism, immune activation, endothelial repair, and extracellular matrix turnover	Multitask learning, network models, feature selection, integrative risk scores	Supports pathway discovery, biomarker prioritisation, and future precision prevention	Cost, batch effects, missing data, limited deployability, risk of overfitting	Minimal external validation in CCS; no regulatory status; hypothesis-generating

Abbreviations: AI-ECG, artificial intelligence-enhanced electrocardiography; CCTA, coronary CT angiography; FFRCT, fractional flow reserve derived from CT; INOCA, ischaemia with non-obstructive coronary arteries; LDL, low-density lipoprotein; PCAT, pericoronary adipose tissue; VSMC, vascular smooth muscle cell.

## Data Availability

Data are available upon reasonable request from the corresponding author.
